# Circulation of polyclonal OXA-244-producing *Escherichia coli* lineages in Jerusalem, Israel

**DOI:** 10.1093/jacamr/dlaf210

**Published:** 2025-11-13

**Authors:** Janko Sattler, Yonatan Oster, Helena M B Seth Smith, Yukino Gütlin, Ayelet Michael-Gayego, Dan Reshef, Violeta Temper, Karsten Borgwardt, Adrian Egli, Jacob Strahilevitz, Jacob Moran-Gilad

**Affiliations:** Department of Machine Learning and Systems Biology, Max Planck Institute of Biochemistry, Martinsried, Germany; Department of Clinical Microbiology and Infectious Diseases, Hadassah-Hebrew University Medical Center, Jerusalem, Israel; Faculty of Medicine, Hebrew University, Jerusalem, Israel; Institute of Medical Microbiology, University of Zurich, Zurich, Switzerland; Institute of Medical Microbiology, University of Zurich, Zurich, Switzerland; Department of Clinical Microbiology and Infectious Diseases, Hadassah-Hebrew University Medical Center, Jerusalem, Israel; Department of Clinical Microbiology and Infectious Diseases, Hadassah-Hebrew University Medical Center, Jerusalem, Israel; Department of Clinical Microbiology and Infectious Diseases, Hadassah-Hebrew University Medical Center, Jerusalem, Israel; Department of Machine Learning and Systems Biology, Max Planck Institute of Biochemistry, Martinsried, Germany; Institute of Medical Microbiology, University of Zurich, Zurich, Switzerland; Department of Clinical Microbiology and Infectious Diseases, Hadassah-Hebrew University Medical Center, Jerusalem, Israel; Faculty of Medicine, Hebrew University, Jerusalem, Israel; Department of Clinical Microbiology and Infectious Diseases, Hadassah-Hebrew University Medical Center, Jerusalem, Israel

## Abstract

**Background and objectives:**

OXA-244-producing *Escherichia coli* represents an emerging concern in Europe due to its rapid spread and difficult-to-detect phenotype. While other OXA-48-like carbapenemases emerged in Israel in 2007, the circulation of OXA-244-producing *E. coli* was only recently reported. We aimed to investigate the proportion of OXA-244 amongst OXA-48-like-producing *E. coli* in a tertiary care university hospital in Jerusalem, Israel, during 2024. We analysed their mode of acquisition, phylogeny, resistome, and the genetic context of blaOXA-244 in these isolates.

**Patients and methods:**

In 2024, 171 patients were identified with OXA-48-like-producing *E. coli* from screening or clinical samples. Of these, 53 were selected using convenience sampling across the entire year for whole genome characterization using short-read sequencing and a subset also underwent long-read sequencing.

**Results:**

Amongst the 53 sequenced OXA-48-like-producing *E. coli*, the majority harboured *bla*_OXA-244_ (*n* = 34), followed by *bla*_OXA-48_ (*n* = 11), and *bla*_OXA-181_ (*n* = 8). Of the 34 OXA-244-producing *E. coli*, transmission was classified as probably or possibly healthcare-associated for 88.2%, and community-acquired for 11.8%. The OXA-244-producing *E. coli* isolates belonged to 13 distinct STs that mainly matched internationally described clones. Core genome MLST demonstrated seven genomic clusters (≤10 allele differences), indicating close common ancestry. Long-read sequencing demonstrated that *bla*_OXA-244_ was chromosomally located within variants of the transposon Tn*51098* across STs.

**Conclusions:**

This study demonstrates the predominance of OXA-244-producing *E. coli* in a tertiary care hospital in Jerusalem, Israel among OXA-48-like-producing isolates. The clonal diversity points to ongoing unrecognized community transmission, necessitating targeted surveillance and control measures.

## Introduction

Carbapenemase-producing *Enterobacterales* (CPE) represent one of the most urgent antimicrobial resistance threats globally.^1^ Among these enzymes, OXA-48-like carbapenemases have emerged as major contributors to carbapenem resistance, with distinct subtypes exhibiting varying geographical distributions and molecular characteristics.^2^

OXA-244, differing from OXA-48 by a single amino acid substitution (Arg214Gly), was first identified in Spain in 2012.^3^ This variant exhibits reduced carbapenem- and temocillin-hydrolysing activity compared to OXA-48. The resulting low carbapenem MICs can lead to missed diagnoses when phenotypic screening methods are used.^4,5^ While for OXA-48-producing *Enterobacterales* there is experimental and clinical evidence that monotherapy with carbapenems is inferior to other therapies, despite carbapenem MICs being in the susceptible range,^6–8^ the impact of OXA-244 production on carbapenem treatment efficacy remains unclear.^9^ Unlike other OXA-48-like variants that typically disseminate via plasmids, the OXA-244 gene, *bla*_OXA-244_, is typically integrated into the chromosome via the transposon Tn*51098*, particularly in *Escherichia coli*, creating a distinct epidemiological pattern.^2^ This chromosomal integration may confer enhanced stability and persistence compared to plasmid-borne variants. Recent surveillance data indicate rapidly increasing OXA-244 prevalence across Europe, with large outbreak clusters identified in several countries and the enzyme emerging as a major OXA-48-like variant of concern.^9,10^

In Israel, early reports on OXA-48-like-producing *Enterobacterales* linked these isolates to medical repatriation from endemic regions.^11–13^ However, autochthonous outbreaks have been increasingly reported since 2016, indicating local establishment, particularly within healthcare facilities.^14^ Despite these developments, the prevalence of specific OXA-48-like subtypes remained largely unexplored until molecular typing recently revealed OXA-244-producing *E. coli* in a hospital outbreak.^15^ This finding raised critical questions about the actual contribution of different OXA-48-like subtypes to the regional epidemiological landscape and the potential for unrecognized OXA-244 circulation.

To address this knowledge gap, we conducted a molecular epidemiological surveillance study of OXA-48-like-producing *E. coli* in a tertiary care centre in Jerusalem during 2024. Our investigation revealed an unexpectedly high prevalence of OXA-244, prompting detailed phylogenetic and genomic analyses to characterize transmission patterns and genetic environments. These findings demonstrate the importance of investigating OXA-244’s prevalence in regions lacking routine OXA-48-like subtyping and highlight implications for surveillance strategies and infection control practices.

## Materials and methods

### Study setting and design

This study was conducted at Hadassah-Hebrew University Medical Centre, a 1100-bed tertiary-care academic hospital with two campuses in Jerusalem, Israel, from January to December 2024. Carbapenemase-producing *Enterobacterales* isolates were identified through patient screening for rectal carriage for several indications: (i) upon admission per risk factors, mainly recurrent admissions and per national guidelines; (ii) contact screening, following epidemiological investigation of CPE acquisition; (iii) in-hospital transfer to high-risk units, such as intensive care units; and (iv) prior to discharge to long-term care facilities.

### Classification of acquisition mode

The acquisition mode of CPE was classified through systematic epidemiological investigations according to standardized definitions (Table 1). Each case was assigned to one of four categories based on the timing of detection and healthcare exposure history.

**Table 1.** Definitions of carbapenemase-producing *Enterobacterales* (CPE) acquisition modes

Mode of acquisition	Definition
Probable in-hospital acquisition (PRIA)	1. Detection of CPE ≥48 h following hospital admission, in a patient without previous CPE carriage, and the patient was screened upon admission and found negative, or
	2. Detection of CPE <48 h following admission in a patient hospitalized at our institute for at least 24 h in the previous month but not in any other acute hospital or long-term care facility (LTCF), and found negative upon screening on that previous admission.
Possible in-hospital acquisition (POIA)	1. Detection of CPE ≥48 h following hospital admission, in a patient without previous CPE carriage, and the patient was not screened upon admission, or
	2. Detection of CPE <48 h following hospital admission, in a patient hospitalized at our institute, including day care, in the last 6 months, but did not fit the PRIA definition, even if they were hospitalized at another medical centre.
Healthcare-associated (HA)	Detection of CPE <48 h following hospital admission, in a patient hospitalized in any other hospital or LTCF in the last 6 months, including day hospitalization, and did not fit the POIA definition.
Community acquisition (CA)	Detection of CPE <48 h following hospital admission, without prior admissions in the previous 6 months.

### Initial screening and characterization

Screening involved rectal swabs cultured using selective chromogenic media (CHROMagar mSuperCARBA, HyLabs, Rehovot, Israel), which allows for the reliable detection of OXA-244-producing *Enterobacterales*.^16^ Species identification of suspected colonies was done using the VITEK MS instrument (bioMérieux, Marcy l’Etoile, France), and confirmation as CPE using a multiplex PCR assay for carbapenemase-encoding gene families (*bla*_OXA-48_, *bla*_NDM_, *bla*_KPC_, *bla*_VIM,_ and *bla*_IMP)_ (EnteroDR, Seegene, South Korea). Clinical isolates recovered from sites of infection during standard diagnostic work-up that were suspected as CPE based on carbapenem MIC values were similarly evaluated.

### Whole genome sequencing and assembly

A representative sample of 53 non-duplicate OXA-48-like-producing *E. coli* isolates was selected for WGS, ensuring temporal distribution covering each month of 2024. DNA extraction was performed using the DNeasy Blood & Tissue Kit (Qiagen, Hilden, Germany) according to the manufacturer’s instructions. The DNA libraries for Illumina sequencing were prepared with the Nextera Flex kit (Illumina, San Diego, CA, USA) or Qiaseq FX library kit (Qiagen, Hilden, Germany) according to the manufacturer’s recommendations, followed by paired-end sequencing on Illumina NovaSeq 6000 (PE150) or NextSeq1000 (PE150) platforms (Illumina, San Diego, CA, USA). Raw sequencing data were quality assessed with FastQC v0.11.9, trimmed with Trimmomatic v0.39, and assembled using SPAdes v3.14.1.

Seven representative isolates (one per major ST) underwent additional long-read sequencing to characterize the genomic environment of *bla*_OXA-244_. DNA was extracted with the Maxwell RSC Blood DNA Kit (Promega, Madison, WI, USA), and libraries were prepared using the Rapid barcoding kit (RBK114.24) (Oxford Nanopore Technologies, Oxford, UK). The DNA was sequenced on a R10.4.1 (FLO-MIN114) flow cell with the GridION sequencer (Oxford Nanopore Technologies, Oxford, UK). Basecalling was performed with Dorado in super-accuracy mode within MinKNOW 24.06.15. Hybrid assemblies were generated using Unicycler v0.4.8.^17^ Details on the raw data and the assemblies are provided in Table S1 (available as Supplementary data at *JAC-AMR* Online).

### Genomic characterization

Antimicrobial resistance genes and plasmid content were identified using the ResFinder database (2023-Nov-4 release)^18^ and the PlasmidFinder database (2023-Jan-18 release)^19^ via ABRicate v1.0.1 (Seemann T, *Abricate*, Github https://github.com/tseemann/abricate.). For *bla*_OXA-48_-like genes, thresholds of 100% identity and coverage were applied, while for all other resistance genes, the thresholds were 98% identity and 90% coverage. Resistome findings for carbapenemase and ESBL genes were confirmed with The Comprehensive Antibiotic Resistance Database (CARD v4.0.1).^20^ For examination of the genomic environments of *bla*_OXA-244_ in the hybrid assemblies, the respective contigs were further investigated. The Tn*51098* variants were annotated with Geneious Prime (Dotmatics, Boston, MA, USA), using publicly available annotated sequences from Emeraud *et al*.^21^ as reference. The other chromosomal regions were annotated from RefSeq *E. coli* reference genomes (GCF_000005845.2 and GCF_000008865.2). Unannotated regions were subsequently interrogated via NCBI nucleotide BLAST to retrieve additional genomes for annotation reference. The different Tn*51098* variants were visualized with clinker.^22^ To assign short read sequences to one of the Tn*51098* variants, short reads were mapped to these variant sequences from the hybrid assemblies with the Geneious Mapper.

### Phylogenetic analysis

Sequence types were determined using the seven-loci Achtman scheme^23^ via EnteroBase.^24^ Core genome MLST (cgMLST) analysis was also conducted using the EnteroBase *E. coli* scheme (2513 loci), and hierarchical clustering was employed to determine phylogenetically closely related isolates.^25^ From these data, a neighbour-joining tree was created with NINJA^26^ and visualized with iTOL.^27^ To identify genomic clusters with close common ancestry, a threshold of ≤10 allelic differences was applied as described in similar studies.^28,29^ These clusters were further analysed with whole genome SNP phylogeny using Snippy v4.6.0 (Seemann T, *Snippy*, Github https://github.com/tseemann/snippy.), except cluster 3, due to sequence contamination in one sample that could not be resolved.

To compare OXA-244-producing *E. coli* from this study to internationally recognized clones, publications were reviewed for relevant outbreaks. Where sequencing data were publicly available, these were included in the comparison, with the selection of one genome per ST per country for practical visualization purposes. To visualize phylogenetic relationships between local and international isolates, a core-genome SNP-based phylogeny was calculated using CSIPhylogeny^30^ and visualized using iTOL.^27^

## Ethical approval

The use of anonymous clinical and microbiological data has been approved by the Research Ethics Committee of the Hadassah Medical Centre, Jerusalem, Israel (#HMO-0019-16).

## Data availability

Genome assemblies of all OXA-244-producing *E. coli* have been deposited on GenBank under Bioproject PRJNA1262528.

## Results

### Epidemiology

During 2024, 32 355 rectal screening swabs from 26 281 patients were processed at the Hadassah Medical Centre Clinical Microbiology Laboratory. Of these, 1116 swabs (3.4%) were positive for any PCR-confirmed CPE isolates. These positive screening samples originated from 683 (screen positivity rate 2.6%) unique patients (multiple samples per patient were common during the study period). Of 683 CPE-carrying patients, 423 (61.9%) carried NDM-producing isolates, 195 (28.6%) carried OXA-48-like-producing isolates, 85 (12.4%) carried KPC-producing isolates, and 22 (3.2%) carried VIM-producing isolates (several patients carried more than one CPE isolate). Of the 195 OXA-48-like-producing CPE isolates, 171 were OXA-48-like-producing *E. coli*, comprising 25.0% of the CPE carriers. The remaining 24 OXA-48-like producers included 15 *Klebsiella pneumoniae* and 9 different *Enterobacter* and *Citrobacter* species. From the 171 OXA-48-like-producing *E. coli*, we selected 53 non-duplicate isolates using convenience sampling across the entire year for genomic characterization via short-read sequencing. Sampling ensured representation of all 12 months without considering epidemiological criteria. Carbapenemase gene analysis revealed that the majority harboured *bla*_OXA-244_ (*n* = 34, 64.2%), followed by *bla*_OXA-48_ (*n* = 11, 20.8%) and *bla*_OXA-181_ (*n* = 8, 15.1%).

Patient epidemiological characteristics are summarized in Table 2 (see Table S1 for individual data). Of the 171 OXA-48-like carriers, 14% were co-colonized with other CPEs. Sixteen carriers (9.4%) had also clinical infections with OXA-48-like-producing *E. coli.* Infection control investigations revealed predominantly healthcare-associated transmissions. Notably, OXA-244 cases were more frequently classified as probable or possible in-hospital acquisitions and less often attributed to other healthcare facilities, suggesting more localized transmission dynamics within the study hospital.

**Table 2.** Epidemiological characteristics of patients with OXA-48-like-producing *Escherichia coli* carriage or infection and of the OXA-244-producing subgroup

	Carriers of *E. coli* OXA-48-like	Carriers of *E. coli* OXA-244
Sample size	*N* = 171	*N* = 34
Mean age	52	46
Male gender	87 (50.9%)	21 (61.8%)
Clinical CPE infection^a^	16 (9.4%)	3 (8.8%)
Previous CPE carriage	23 (13.5%)	4 (11.8%)
Co-colonization with additional CPE types	24 (14.0%)	6 (17.6%)
Hospitalized in the previous 6 months	130 (76.0%)	29 (85.3%)
Mode of acquisition		
Probable in-hospital acquisition	42 (24.6%)	8 (23.5%)
Possible in-hospital acquisition	77 (45.0%)	20 (58.8%)
Healthcare associated	25 (14.6%)	2 (5.9%)
Community acquisition	27 (15.8%)	4 (11.8%)

^a^All positive cases had a clinical infection involving OXA-48-like *E. coli*.

### Phylogeny and resistome of OXA-244-producing *E. coli*

**Figure 1.**
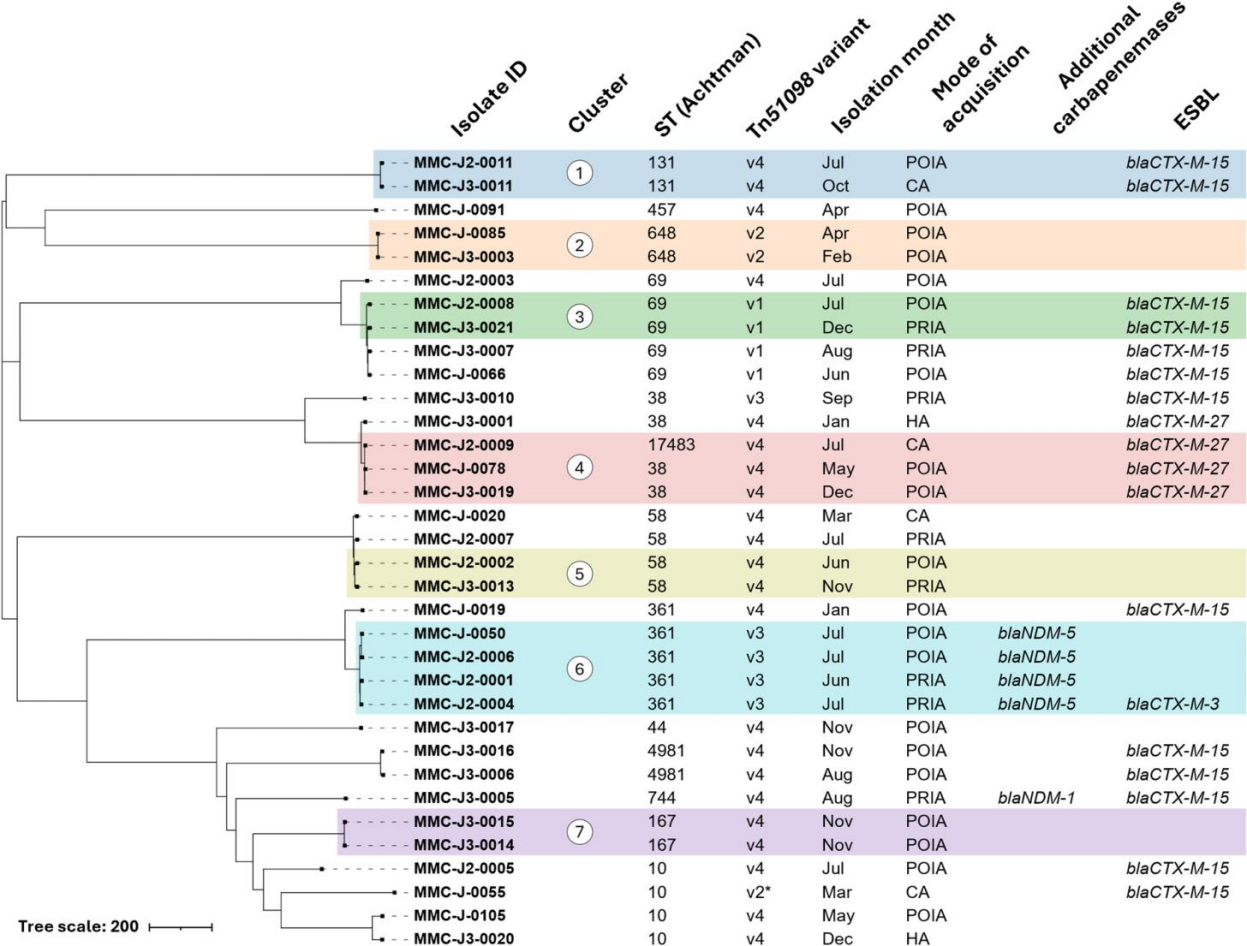
Allele-based neighbour-joining tree of OXA-244-producing *Escherichia coli* from 2024, constructed from cgMLST profiles using the EnteroBase scheme. Branch lengths represent allelic differences. Colours indicate clusters of isolates differing by up to 10 alleles. Metadata columns (isolate ID, ST, Tn*51098* variant, month of isolation, mode of acquisition, resistance genes) are shown alongside the tree. A detailed description of Tn*51098* variants is given in section ‘Genetic Environment of *bla*_OXA-244_’. Asterisk indicates Tn*51098* variant 2 with a minor truncation. PRIA: Probable in-hospital acquisition, POIA: Possible in-hospital acquisition, HA: Healthcare associated, CA: Community acquisition. Figure produced with iTOL.

Analysis of the 34 OXA-244-producing *E. coli* isolates revealed remarkable genetic diversity, with 13 distinct STs (Figure 1). Core genome MLST analysis identified seven genomic clusters (threshold  ≤ 10 allelic differences), with isolation dates spanning up to 8 months within individual clusters. Among these clusters, whole genome SNP analysis indicated that clusters 2, 4, and 7 were consistent with recent transmission, with SNP differences of 4, 5–11, and 3, respectively (Table S2). The same was true for isolates MMC-J-0050 and MMC-J2-0006 from cluster 6 with 8 SNPs difference. All other isolates showed pairwise SNP differences of at least 20 SNPs (range 20–43), making very recent transmission events less likely.

Following an in-depth review of spatio-temporal hospital data, links were identified for three of the seven clusters, including contact between two patients in a surgical ward (cluster 2), contact between two patients in another surgical ward (cluster 5), and two patients in a medical ward (cluster 6, isolates MMC-J2-0001 and -0004). Interestingly, no links were identified for the two other cases in this cluster.

The resistome analysis demonstrated the presence of different resistance genes beyond OXA-244 (Figure 1; Table S1). Co-occurring carbapenemases were detected in 14.7% of isolates (four with *bla*_NDM-5_, one with *bla*_NDM-1_), while ESBL genes were present in over half of the collection (52.9%), predominantly *bla*_CTX-M-15_ (38.2% of total isolates) (Figure 1). The resistance gene profiles of carbapenemase and ESBL genes generally corresponded with phylogenetic clustering, supporting a close relationship in 16/17 clustered isolates.

Phylogenetic comparison with representative international OXA-244-producing *E. coli* revealed that the local strain population generally corresponds with global STs (Figure S1). Of note, five STs found in the current study (ST44, ST457, ST744, ST4981, and ST17483) represent novel associations with OXA-244.

### Genetic environments of *bla_OXA-244_*

**Figure 2.**
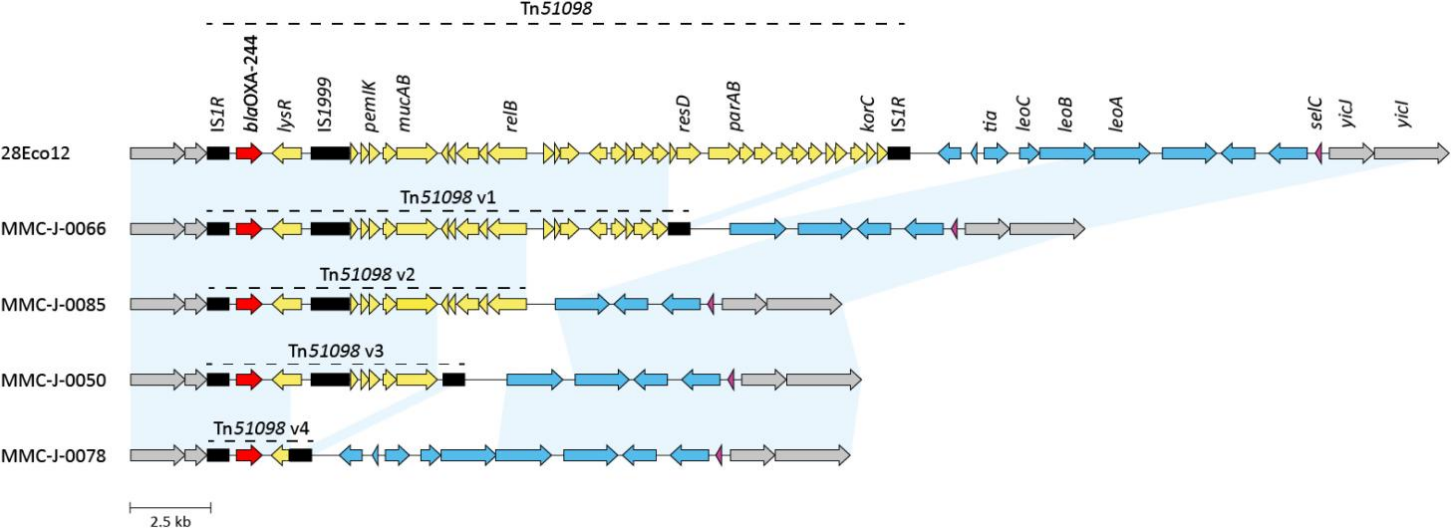
Variants of the transposon Tn*51098* and the surrounding genetic environment found in OXA-244-producing *Escherichia coli* isolates in this study. Reference was the 28Eco12 isolate with a full Tn*51098* transposon described by Abril et al. (2019). Red = bla_OXA-244_, black = insertion sequences, yellow = other open reading frames (ORFs) within Tn*51098*, purple = *selC*, blue = ORFs in the variable region between *selC* and Tn*51098*, grey = ORFs outside of the abovementioned regions. Figure produced with clinker.

Long-read sequencing of seven representative isolates spanning distinct STs revealed universal chromosomal integration of *bla*_OXA-244_ within truncated variants of transposon Tn*51098*. These seven assemblies defined four distinct Tn*51098* structural variants, which served as references for classifying the genetic context in all 34 isolates. Short-read mapping of the remaining 27 OXA-244-producing isolates demonstrated that each could be assigned to one of these four variants (Figure 1), with one isolate (MMC-J-0055) showing a minor truncation of the *relB* gene (288 bp) within Tn*51098* variant 2. Tn*51098* variant 4 was most frequently detected (*n* = 22) and comprised only the flanking IS*1R* elements, the *bla*_OXA-244_ gene, and a truncated *lysR* gene (Figure 2). The remaining variants were less common: variant 3 (*n* = 5), variant 1 (*n* = 4), and variant 2 (*n* = 3). Transposon variant distribution correlated strongly with phylogenetic clustering, reinforcing evidence for clonal transmission of specific lineages.

In the seven isolates that underwent long-read sequencing, Tn*51098* insertion occurred in the chromosome within a genomic island inserted in the *selC* gene. Tn*51098* variants 1, 3, and 4 formed composite transposons bounded by IS*1R* elements with inverted repeats. These transposons were flanked by direct repeats. In the Tn*51098* variant 2, the downstream IS*1R* element was absent, along with its corresponding direct repeat (Figure 2).

While the upstream genomic region (relative to *bla*_OXA-244_) remained consistent across all long-read sequenced isolates, the downstream region between Tn*51098* and *selC* showed several variations. Tn*51098* variant 4 matched the downstream structure described in the reference Tn*51098,* while Tn*51098* variants 1 and 3 lacked genes between IS*1R* and *leoB*, and variant 2 additionally lacked the *leoB* gene (Figure 2).

## Discussion

### OXA-244 is the predominant variant of OXA-48-like in clinical *E. coli* isolates in Jerusalem, Israel

This study demonstrates the high prevalence of OXA-244-producing *E. coli* in a large centre in Israel, representing a significant shift from historical epidemiological patterns. Since the initial detection in 2007 of OXA-48-like carbapenemases in *E. coli* in Israel,^11^ reports have until recently largely described sporadic cases or localized outbreaks rather than endemic circulation. The lack of routine OXA-48-like subtyping in earlier studies may have masked the contribution of OXA-244 to the resistance landscape until molecular characterization recently revealed its involvement in hospital outbreaks.^15^

Our finding that OXA-244 comprises 64.2% of the sequenced OXA-48-like-producing *E. coli* in this hospital during 2024 is particularly significant given that this variant was previously considered uncommon (Pitout et al. 2019). Notably, the detection of OXA-244-producing isolates strongly depends on the screening agar type used^16^ and, therefore, the reported prevalence might have previously been underestimated when agar types with lower sensitivity were used. The high prevalence of OXA-244 aligns with a nationwide study in Isreal, that identified OXA-244 as the most common subtype amongst OXA-48-like-producing *E. coli* between 2021 and 2023 in Israel.^31^ It furthermore aligns with broader European trends where OXA-244 has rapidly emerged since 2018 to become the dominant carbapenemase in *E. coli* across multiple countries.^9,10,32^ Such high prevalence rates indicate established endemicity and underscore the critical need for enhanced surveillance incorporating molecular subtyping of OXA-48 variants.

### International connectivity and regional circulation patterns of OXA-244-producing *E. coli*

Frequent epidemiological connections have been documented between OXA-244-producing *E. coli* detections in Europe and international travel, particularly to Egypt, Turkey, and other Middle East and North Africa (MENA) regions.^5,10,33,34^ These travel-associated cases suggest that OXA-244 is endemic within MENA countries, with international travellers serving as vehicles for strain introduction into Europe. However, epidemiological data on OXA-244-producing *E. coli* from the MENA region are scarce. Current reports from neighbouring countries describe only isolated findings: individual clinical isolates from Egypt^35^ and Qatar,^36^ and environmental detections from Algeria^37^ and Lebanon.^38^ Our phylogenetic analysis reveals that Israeli strains also share close evolutionary relationships with European isolates, reflecting Israel's strong connectivity with Europe through tourism, immigration, and commerce.

Large-scale molecular epidemiological studies across the region would be needed to determine the true prevalence of OXA-244 circulation and clarify the directionality and mechanisms of international strain movement.

### Mode of transmission is mainly healthcare-associated, but polyclonality indicates circulation in the community

In our cohort of OXA-244-producing *E. coli*, 88.2% of cases were classified as probable or possible in-hospital transmission or other healthcare-associated acquisition. This high rate of healthcare association somewhat exceeds the 72.5% reported in the Temkin *et al*. nationwide study,^31^ likely reflecting our urban tertiary care setting and possibly deeper case interrogation. However, the remarkable genetic diversity—13 distinct STs among only 34 isolates with some isolates showing more than 2000 cgMLST allelic differences—argues against purely nosocomial circulation. These data suggest that healthcare facilities are receiving strains from diverse reservoirs, such as the community and potentially international movement, rather than generating diversity through sustained hospital spread alone. Importantly, the mode of acquisition classification was purely based on the infection control scheme shown in Table 1. However, combining in-depth investigation of hospital data with available genomic data, we identified only one case where epidemiological patient movement investigations and SNP-based phylogenetic analysis aligned perfectly to demonstrate recent direct transmission. This finding underscores that both the epidemiological investigation and genomic analysis are complementary approaches required for outbreak detection and strain tracking, though our sequencing of only 53 of the 171 OXA-48-like isolates may have limited our ability to detect additional transmission clusters. The important role of strain importation from outside the hospital is further supported by the finding that some community-acquired isolates cluster phylogenetically with hospital-acquired strains, suggesting possible bidirectional exchange between healthcare and community settings. The epidemiological classification scheme used here likely underestimates true community circulation. The standard 6-month healthcare exposure window may misclassify patients who acquired OXA-244-carrying *E. coli* in the community earlier but had recent unrelated healthcare contact, given that OXA-244 carriage can persist for over 3 years.^34^ Furthermore, phylogenetic clusters span multiple hospitals across Israel, supporting regional rather than institution-specific circulation.^31^ This pattern suggests that community reservoirs contribute to OXA-244 dissemination in Israel, as described before in European settings,^39^ thus highlighting the need for community-focused surveillance and prevention strategies.

### Genetic environment of *bla_OXA-244_*

Our findings demonstrate universal chromosomal integration of *bla*_OXA-244_ within Tn*51098* variants across all seven isolates examined with long-read sequencing. While all other OXA-48-like variants disseminate primarily through plasmid transfer, OXA-244 consistently utilizes chromosomal integration via transposon-mediated mechanisms, with Tn*51098* and its truncated derivatives serving as the exclusive genetic vehicles described to date.^21^ The only comparable pattern within the OXA-48-like group is the chromosomal integration of *bla*_OXA-48_ in *E. coli* ST38 through transposon Tn*6237*. This phenomenon is predominantly restricted to a single ST and has been linked to the antiplasmid system *apsAB*.^40^ In contrast, our data demonstrate that *bla*_OXA-244_ successfully integrates chromosomally across diverse STs, indicating a distinct driver for chromosomal integration. This is further supported by the absence of *apsAB* genes in all OXA-244-producing isolates of this study. This exceptional integration versatility across multiple lineages suggests adaptive advantages and may explain OXA-244's epidemiological success. The transposon-mediated chromosomal integration likely confers enhanced genetic stability, reduced fitness costs, and improved growth rates compared to plasmid-carrying isolates.^40^ The molecular mechanisms enabling such broad host compatibility warrant investigation to understand how OXA-244 has evolved this remarkable capacity for cross-lineage chromosomal establishment, and whether this capacity had possibly emerged from ancestral strains of OXA-48-producing *E. coli*.

### Limitations

This study has several limitations. First, our analysis focused on a single medical centre over one year, limiting the generalizability of findings to broader regional epidemiology or different time periods, despite the substantial dataset generated. Second, our sampling of 53 from 171 OXA-48-like-producing *E. coli* isolates may not fully represent the genetic diversity and resistance mechanisms present in the complete collection, potentially affecting our understanding of circulating strain populations and transmission dynamics. Third, OXA-244 may go undetected during routine surveillance due to its characteristically low carbapenem MICs compared to other carbapenemases,^5^ potentially resulting in an underestimation of its true prevalence.

### Conclusions

In summary, this study demonstrates widespread establishment of genetically diverse OXA-244-producing *E. coli* in a major hospital in Jerusalem, Israel, indicating healthcare-associated transmission coupled with substantial community circulation. These findings highlight the need for enhanced surveillance strategies extending beyond acute care facilities. Our data also demonstrate that *bla*_OXA-244_ successfully integrates chromosomally across diverse STs through transposon-mediated integration, likely conferring selective advantages. The mechanisms enabling such broad host compatibility warrant further investigation.

## Supplementary Material

dlaf210_Supplementary_Data

## Supplementary data

Figure S1 and Tables S1 and S2 are available as Supplementary data at JAC-AMR Online.
