# Supplementary material for: Circulation of polyclonal OXA-244-producing Escherichia coli lineages in Jerusalem, Israel
Source: JAC Antimicrob Resist. 2025 Nov 13;7(6):dlaf210. doi: 10.1093/jacamr/dlaf210 (PMC12611639; doi:10.1093/jacamr/dlaf210)
Supplement: dlaf210_Supplementary_Data [file dlaf210_supplementary_data.zip › Supplementary_Materials_S2_20250919_2_JS.docx]

**Circulation of polyclonal OXA-244-producing *Escherichia coli* lineages in Jerusalem, Israel**

***Supplementary Materials***

**Supplementary Table S2:** Pairwise SNP distance matrices within genomic clusters based on whole genome SNP analysis of raw reads. One assembly from each cluster was used as the reference genome. Cluster 3 could not be analyzed due to sequence contamination that could not be resolved.

| **Cluster 1** | MMC-J2-0011 | MMC-J3-0011 |  |  |
| --- | --- | --- | --- | --- |
| MMC-J2-0011 | 0 | 30 |  |  |
| MMC-J3-0011 | 30 | 0 |  |  |
|  |  |  |  |  |
| **Cluster 2** | MMC-J-0085 | MMC-J3-0003 |  |  |
| MMC-J-0085 | 0 | 4 |  |  |
| MMC-J3-0003 | 4 | 0 |  |  |
|  |  |  |  |  |
| **Cluster 4** | MMC-J-0078 | MMC-J2-0009 | MMC-J3-0019 |  |
| MMC-J-0078 | 0 | 11 | 12 |  |
| MMC-J2-0009 | 11 | 0 | 5 |  |
| MMC-J3-0019 | 12 | 5 | 0 |  |
|  |  |  |  |  |
| **Cluster 5** | MMC-J2-0002 | MMC-J3-0013 |  |  |
| MMC-J2-0002 | 0 | 43 |  |  |
| MMC-J3-0013 | 43 | 0 |  |  |
|  |  |  |  |  |
| **Cluster 6** | MMC-J-0050 | MMC-J2-0001 | MMC-J2-0004 | MMC-J2-0006 |
| MMC-J-0050 | 0 | 33 | 29 | 8 |
| MMC-J2-0001 | 33 | 0 | 20 | 33 |
| MMC-J2-0004 | 29 | 20 | 0 | 29 |
| MMC-J2-0006 | 8 | 33 | 29 | 0 |
|  |  |  |  |  |
| **Cluster 7** | MMC-J3-0014 | MMC-J3-0015 |  |  |
| MMC-J3-0014 | 0 | 3 |  |  |
| MMC-J3-0015 | 3 | 0 |  |  |


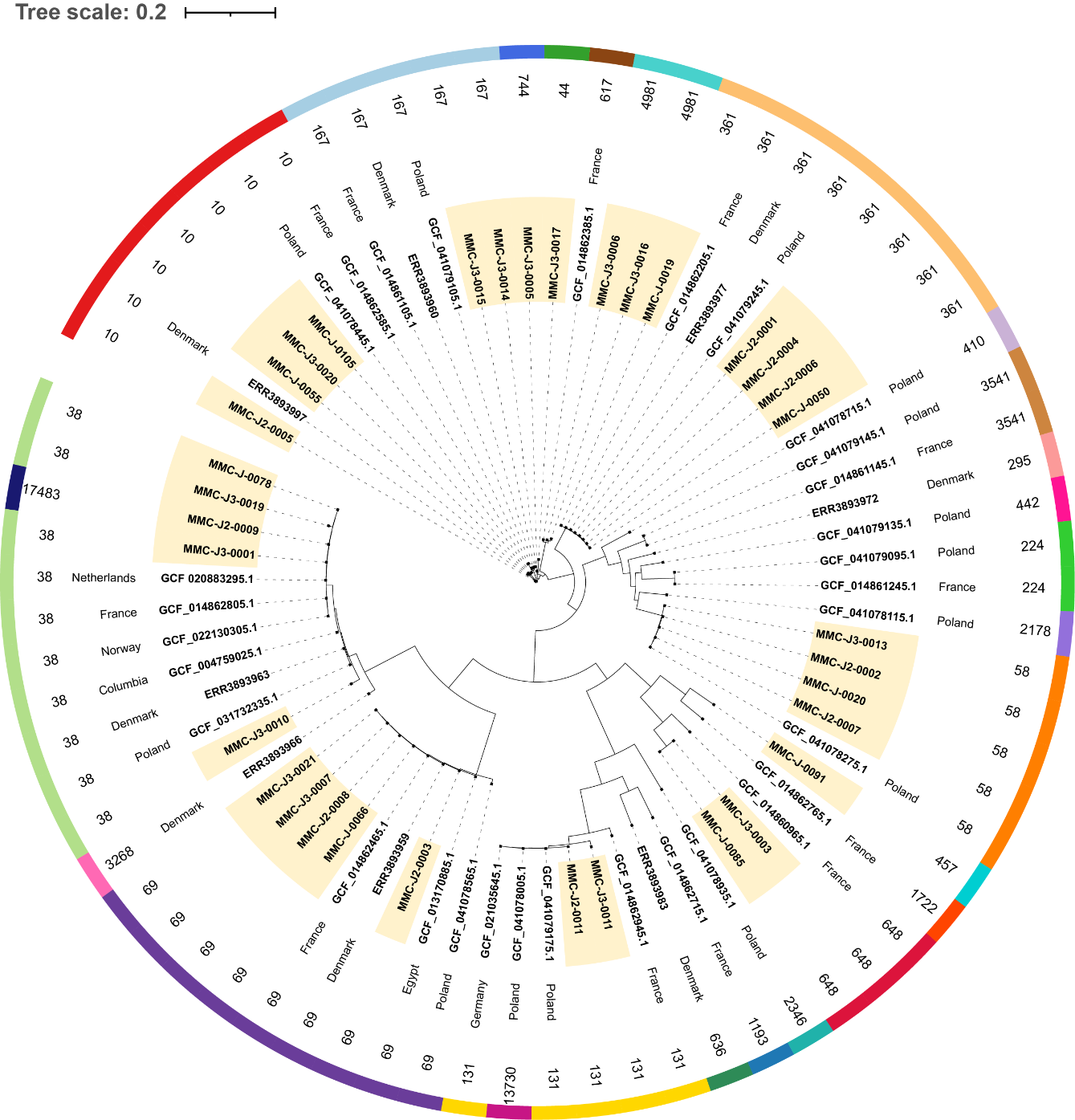


**Supplementary Figure S1:** Whole genome SNP-based phylogenetic tree of OXA-244-producing *Escherichia coli* from this study (isolate ID highlighted in light yellow) and representative reference strains from public repositories with country of origin. Sequence types are coloured as per group. Figure produced with iTOL.
